# Buriti (*Mauritia flexuosa* L.f.) and Acuri (*Attalea phalerata* Mart. ex Spreng) Oils as Functional Lipid Sources in Bakery Products: Bioactive Composition, Sensory Evaluation, and Technological Performance

**DOI:** 10.3390/foods14173089

**Published:** 2025-09-02

**Authors:** Renata Nascimento Matoso Souto, Jorge da Silva Pinho, Carolina Lírio Didier Peixe, Maria Eduarda Flores Trindade, Pâmela Gomes de Souza, Pítias Eduardo da Silva, Bárbara Elisabeth Teixeira-Costa, Vanessa Naciuk Castelo-Branco, Anderson Junger Teodoro

**Affiliations:** 1 Postgraduate Program in Food and Nutrition (PPGAN), Federal University of the State of Rio de Janeiro (UNIRIO), Rio de Janeiro 22290-240, Brazil; renatamatoso@edu.unirio.br; 2 Integrated Food and Nutrition Center (CIAN), Faculty of Nutrition, Fluminense Federal University (UFF), Niterói 24020-140, Brazilcarolinalirio@id.uff.br (C.L.D.P.); mariaeduuardaflores@gmail.com (M.E.F.T.); betcosta@id.uff.br (B.E.T.-C.); 3 Postgraduate Program in Applied Health Products (PPGCAPS), Faculty of Pharmacy, Fluminense Federal University (UFF), Niterói 24241-000, Brazil; 4 Department of Food Science and Nutrition, Faculty of Food Engineering, State University of Campinas—UNICAMP, Campinas 13083-970, Brazil; pgdsouza.pharma@gmail.com; 5 Chemical Institute, Fluminense Federal University (UFF), Niterói 24020-007, Brazil; pitiases@id.uff.br; 6 Postgraduate Program in Biotechnology, Federal University of Amazonas, Manaus 69077-000, Brazil; 7 Department of Bromatology, Faculty of Pharmacy, Fluminense Federal University (UFF), Niterói 24241-000, Brazil; vanessanaciuk@id.uff.br

**Keywords:** native fruits, fat-soluble bioactive, bread

## Abstract

Given the growing consumer demand for improved quality of life and health-promoting foods, replacing conventional fats in widely consumed products such as bread with oils derived from native Brazilian fruits represents a promising strategy. This study aimed to evaluate the bioactive and technological potential of buriti (*Mauritia flexuosa*) and acuri (*Attalea phalerata*) oils, extracted from palm fruits native to the Cerrado and Amazon biomes. Both oils proved to be rich sources of lipophilic bioactives, particularly carotenoids, tocopherols, and phenolic compounds, and exhibited excellent carotenoid bioaccessibility under in vitro digestion, with recovery rates of 74% for acuri oil and 54% for buriti oil. Notably, buriti oil showed a high β-carotene content (1476.5 µg/g). When incorporated into sandwich bread formulations, these oils enhanced antioxidant activity, improved texture, volume, and color, and maintained high sensory acceptance compared to bread made with soybean oil. Sensory evaluation scores averaged above 7 for all tested attributes. These findings underscore the industrial applicability of buriti and acuri oils as functional lipids aligned with sustainable development and nutritional innovation.

## 1. Introduction

Brazil harbors approximately 25% of the world’s biodiversity, with native fruits from biomes such as the Cerrado and Amazonia standing out for their high agro-industrial potential. The buriti (*Mauritia flexuosa* L.f.) and acuri (*Attalea phalerata* Mart. ex Spreng) palms, both native species from the Arecaceae family, are key resources for regional bioeconomy, primarily due to the high oil content found in their pulps. These oils have broad applications in the food, cosmetic, and pharmaceutical industries [1,2]. In addition to adding socioeconomic value to traditional communities, their production promotes ecosystem conservation and aligns with Sustainable Development Goal (SDG) No. 8 of the 2030 Agenda, which emphasizes strengthening local economies through job creation and agricultural diversification [3,4].

Traditionally, oils extracted from buriti and acuri pulps are obtained by cold mechanical pressing or through artisanal methods involving the heating of the pulp in water until the oil is released [5]. Both methods are considered more sustainable than solvent-based extraction and are consistent with SDG 12 (Responsible Consumption and Production) due to their minimal environmental impact [3]. Recent studies have shown that buriti and acuri oils yield high extraction rates and present a favorable lipid profile, predominantly composed of unsaturated fatty acids such as oleic acid (47.5% in buriti and 47.6% in acuri) [6,7]. Moreover, these oils are rich in carotenoids (e.g., β-carotene and α-carotene) and tocopherols, bioactive compounds widely recognized for their antioxidant and anti-inflammatory properties [8,9,10].

Buriti oil is particularly notable for its high content of total carotenoids and tocopherols, which are associated with anti-inflammatory, antimicrobial, and wound-healing properties [6,11,12], while acuri oil has demonstrated protective effects against cellular damage [7]. These functional properties are in line with the growing consumer demand for healthier foods, driven by market trends that value natural and functional ingredients [13].

Beyond their nutritional value, fats play critical roles in food processing, directly influencing the quality of the final product by affecting its physical, textural, and sensory attributes. These factors impact overall palatability and, consequently, consumer acceptance [14]. Bread is a globally consumed staple food, widely accessible and highly accepted by consumers, and is deeply embedded in the cultural identity of various populations [15]. Moreover, it offers considerable potential for nutritional enhancement through formulation adjustments. In this context, replacing hydrogenated or animal fats, both recognized as sources of trans fats linked to cardiovascular health risks, with oils derived from native fruits of Brazilian biomes represents a promising strategy for the development of healthier bread products [14,16].

The incorporation of buriti and acuri oils into food products, particularly baked goods, may improve functional attributes, including a healthier lipid profile and increased antioxidant capacity. Additionally, their saturated fatty acid content can positively influence technological characteristics such as dough plasticity and crumb softness. These benefits respond to the food industry’s demand for ingredients that combine health benefits with the sustainable use of Brazil’s biodiversity [17,18].

Despite their promising potential, knowledge gaps remain regarding the nutritional and technological roles of these oils in food systems. Accordingly, the objectives of this study were to characterize buriti and acuri oils, with an emphasis on their high content of lipophilic bioactive compounds, antioxidant activity, and carotenoid bioaccessibility, and to evaluate their application in the formulation of sandwich bread.

## 2. Materials and Methods

### 2.1. Raw Materials and Oil Extraction

Cold-pressed buriti oil was supplied by the Grande Sertão Cooperative (Minas Gerais, Brazil) with a yield of 2% from the whole fruit, according to the producer. Acuri oil was obtained via cold pressing at the Food Analysis Laboratory (LABAL) of the Fluminense Federal University, using fruits manually harvested in the municipality of Porto Acre, Acre, Brazil (9°56′51′′ S, 67°49′07′′ W). The fruits were sanitized in a 150 ppm chlorinated solution and manually pulped into flakes. These flakes were dried in a ventilated oven at 60 °C for 3 h and subsequently subjected to cold extraction in a continuous press (Estufa Pardal) to obtain crude acuri oil. The extracted oil was centrifuged at 3000 rpm for 10 min using a centrifuge (Centribio mod 80-2B), then stored under refrigeration in amber bottles until further use. The extraction of oil from whole acuri fruit resulted in a yield of 3.7%.

### 2.2. Fatty Acid Profile by GC-FID

The fatty acid profile of the oil samples was analyzed in triplicate using capillary gas chromatography (GC) of fatty acid methyl esters (FAMEs), which were obtained through direct transesterification of total lipids [19]. A 1.0 µL aliquot of the derivatized sample in n-hexane was injected into a gas chromatograph (GC-2014, Shimadzu^®^, Kyoto, Japan) equipped with a flame ionization detector (GC-FID), a split/splitless injector set to a 1:20 split ratio, and an Omegawax-320 column (30 m × 0.25 mm i.d., 0.25 µm film thickness; Supelco, Co., Bellefonte, PA, USA). The oven was programmed to maintain the initial temperature of 170 °C for 3 min, increasing 1 °C/min until reaching 225 °C, and holding it at that temperature for 5 min. Helium was utilized as the carrier gas at a constant pressure of 100 kPa, while the injector and FID were maintained at 260 °C and 280 °C, respectively. FAMEs were identified based on their relative retention times compared to a commercial standard mixture. Fatty acid quantification was performed via internal normalization of peak areas, adjusted using theoretical correction factors, and expressed as g/100 g of total fatty acids [20].

### 2.3. Total Phenolic Content and Phenolic Profile

The total phenolic content was determined using the Folin–Ciocalteu method adapted to microplate format, as described by Abreu et al. (2019) [21]. Sample absorbance was measured at 750 nm using a microplate spectrophotometer (SpectraMax i3x multi-mode reader, Fremont, CA, USA). A gallic acid standard curve was used for calibration, and the results were expressed as micrograms of gallic acid equivalents (GAE) per milliliter of oil sample. Phenolic compounds were extracted from the oils by liquid–liquid extraction with 80% methanol (*v*/*v*) following dissolution in HPLC-grade hexane (1:2, *w*/*v*). The extraction was performed three times, and the combined methanolic phases were evaporated to dryness. The residue was resuspended in 3 mL of methanol, filtered, and injected into the HPLC system equipped with photodiode array detection (HPLC-PDA) for phenolic profiling. The HPLC system (Shimadzu, Kyoto, Japan) consisted of a quaternary pump (LC-20AT), system controller (CBM-20A), degasser (DGU-20A5), and PDA detector (SPD-M20A). Chromatographic separation was carried out on a reverse-phase silica column (C18, 4.6 mm i.d. × 150 mm, 5 µm particle size; Kromasil^®^, Nouryon, Göteborg, Sweden). The mobile phase consisted of a gradient of 0.3% aqueous formic acid (eluent A), methanol (eluent B), and acetonitrile (eluent C), delivered at a flow rate of 1.0 mL/min under the following conditions: 24% B at 8 min, 28% B at 18 min, 33% B at 30 min, and 65% B at 60 min, followed by a 15 min re-equilibration period. Eluent C was kept constant at 1% throughout the analysis. The concentration of each phenolic compound was expressed in mg per 100 g of oil.

### 2.4. Antioxidant Activity

The antioxidant activity of buriti and acuri oils was evaluated using the DPPH and ABTS radical scavenging assays, with all tests performed in triplicate. The methodologies were adapted from Re et al. [21] and Abreu et al. [22]. For the antioxidant activity assays using the ABTS and DPPH methods, methanolic extracts obtained for the analysis of the phenolic profile were used. For the antioxidant activity and total phenolic content assays of the bread samples, the same methodologies were applied using 1:2 aqueous extracts. For the antioxidant activity assays and the determination of total phenolic content in the bread samples, the same methodologies were applied using aqueous extracts prepared at a 1:2 ratio, consisting of one part of ground bread sample and two parts of ultrapure water.

### 2.5. Tocols Profile

Tocols were analyzed in triplicate using normal-phase high-performance liquid chromatography (HPLC), as described by Silva et al. [23], employing the HPLC system (Shimadzu^®^, Kyoto, Japan) previously detailed in Section 2.5. Oil samples were dissolved in n-hexane, centrifuged (2700× *g* for 5 min), and filtered through a PTFE syringe filter (0.45 μm). A volume of 10 µL of either the standard solutions or the sample was injected into a normal-phase column (250 × 4.6 mm, 5 µm, ZORBAX Rx-Sil; Agilent Technologies, Santa Clara, CA, USA), with a binary isocratic mobile phase composed of n-hexane/2-propanol (99:1, *v*/*v*) at a flow rate of 1.0 mL/min. Commercial standards of tocopherols were used for identification and quantification by external calibration. Tocotrienols were quantified based on the external calibration curve of α-tocotrienol. The concentrations of the tocol stock solutions were determined spectrophotometrically after appropriate dilutions, using the following specific extinction coefficients in ethanol (λ nm; E1% 1 cm): α-tocopherol (292 nm; 75.8), β-tocopherol (296 nm; 89.4), γ-tocopherol (298 nm; 91.4), δ-tocopherol (298 nm; 87.3), and α-tocotrienol (292 nm; 75.8). Calibration curves ranged from 0.5 to 3.0 μg/mL and showed coefficients of determination (R^2^) greater than 0.99. The concentration of each tocol was expressed as μg/g of oil.

### 2.6. Carotenoids Profile

Carotenoids were analyzed by reverse-phase high-performance liquid chromatography with diode array detection (RP-HPLC-DAD), following the methodologies described by Rodriguez-Amaya [24] and O’Sullivan et al. [25]. The HPLC system (Shimadzu^®^, Kyoto, Japan) was equipped with a quaternary pump (LC20AT), diode array detector (SPD-M20A), degasser (DGU-20A5R), autosampler (SIL-20AC), and system controller (CMB20A). For carotenoid extraction, 0.5 g of the sample was diluted in an ethanol/acetone/water solution (11:66:22; *v*/*v*) and mixed with 2.0 mL of HPLC-grade n-hexane, followed by vortexing for 10 s. The upper phase was collected, and the procedure was repeated until the residue became colorless. The collected extracts were combined and evaporated under vacuum at 35 °C using a rotary evaporator. The resulting dry extract was resuspended in 1.0 mL of n-hexane, filtered, and injected into the HPLC system.

Chromatographic separation of the carotenoids was carried out using a reverse-phase C30 column (150 × 4.6 mm, 5 µm, Acclaim™ C30, Thermo Fisher Scientific, Waltham, MA, USA). The mobile phase consisted of a gradient elution of acetonitrile (eluent A), methanol (eluent B), ethyl acetate (eluent C), and 200 mM acetic acid in Milli-Q water (eluent D), with a constant flow rate of 1.5 mL/min. The concentration of eluent D was maintained at 0.5% throughout the analysis. The gradient elution program was as follows: 7.25% of eluents B and C at 0 min, increasing to 17.25% at 25 min, returning to 7.25% at 35 min, followed by a 5 min re-equilibration, resulting in a total run time of 40 min. Carotenoids were monitored at 450 nm, and identification was performed by comparing retention times and UV spectra with those of authentic standards. Quantification was conducted using external calibration curves ranging from 5.0 to 70.0 µg/mL for all carotenoids analyzed. The content of each carotenoid was expressed as µg/g of oil.

### 2.7. Bioaccessibility of Carotenoids

Oil samples were subjected to an in vitro simulated digestion protocol encompassing oral, gastric, and duodenal phases, following the INFOGEST 2.0 protocol adapted for high-fat matrices [8]. A blank assay was performed using distilled water, and all reaction vessels were sealed with silicone septa, replacing atmospheric air with N_2_ gas to maintain an inert atmosphere. To prevent carotenoid degradation, all simulated digestion steps were conducted under dark conditions. Briefly, 0.5 g of each sample was mixed with 4.5 mL of distilled water and vortexed for 30 s. The tubes were then incubated at 37 °C to initiate the oral phase of digestion. During this phase, simulated salivary fluid, prepared according to the INFOGEST protocol, was mixed with the sample at a 1:1 (*w*/*w*) ratio in glass vials and agitated at 37 °C for 2 min [26]. Subsequently, the mixture was acidified with 5.0 M HCl to pH 3.0, followed by the addition of 0.15 mM CaCl_2_, simulated gastric fluid (1:1, *v*/*v*), pepsin (2000 U/mL), and gastric lipase (60 U/mL). The gastric digestion phase was carried out by incubating the mixture under agitation at 37 °C for 2 h.

Following gastric digestion, the mixture was neutralized to pH 7.0 with 5.0 M NaOH, supplemented with 0.6 mM CaCl_2_, simulated duodenal fluid (1:1, *v*/*v*), pancreatin (100 U/mL), pancreatic lipase (2000 U/mL), and bile extract (10 mM). Duodenal digestion proceeded via incubation with agitation at 37 °C for 2 h. All digestive fluids and enzymes were injected into the vials through the silicone septa to maintain the nitrogen atmosphere. The experiments, comprising 120 min of duodenal digestion, were performed in duplicate to independently assess carotenoid bioaccessibility. Upon completion of the duodenal phase, samples were cooled below 10 °C to inactivate enzymes, centrifuged at 4000× *g* for 60 min, and sequentially filtered through Whatman No. 1 filter paper and a 0.45 μm PTFE membrane to isolate the micellar fraction. For carotenoid extraction, the filtrate was transferred to a separatory funnel, sequentially mixed with 10 mL of ethanol/acetone/water (11:66:22, *v*/*v*) and 5.0 mL of n-hexane. After agitation and phase separation, the upper organic phase was collected [24,25]. The aqueous phase was re-extracted using the same procedure until color depletion. The combined extracts were concentrated under vacuum at 35 °C using a rotary evaporator. The dried extracts were resuspended in 0.5 mL of n-hexane, filtered through a 0.45 μm PTFE membrane, and stored at −20 °C under nitrogen in amber glass vials with screw caps until HPLC analysis, performed as described in Section 2.6.

*Bioaccessibility* was calculated using Equation (1):

*Bioaccessibility* (%) = (*Cdig*/*Cundig*) × 100 (1)

where *Cdig* represents the carotenoid concentration in samples subjected to 120 min of duodenal digestion, and *Cundig* is the carotenoid concentration in undigested samples.

### 2.8. Bread Formulation

Buriti oil and acuri oil were employed as lipid ingredients in the preparation of bread, with variations in the amounts added. The bread formulation followed the methodology proposed by Gutkoski and Jacobsen [27], with adaptations, using commercial white wheat flour containing 10% protein (100%), water (53–57%), vegetable oil (7%), refined sugar (4%), dry yeast (1.7%), and salt (2%). The base formulation used in the experiment was derived by modifying the percentage of soybean oil from the original formulation, with substitution levels of buriti oil (BB) or acuri oil (AB) at 0% (control), 25% (BB25 and AB25), 50% (BB50 and AB50), 75% (BB75 and AB75), and 100% (BB100 and AB100). Bread samples were prepared using an automatic bread maker (Mondial brand), with a total processing time of 2 h and 53 min, encompassing kneading, resting, fermentation, and baking, yielding bread weighing approximately 900 g. The proposed formulations, expressed in mass (g) with substitutions of the lipid raw material, are presented in Table 1.

#### 2.8.1. Sensory Analysis

A total of eighty-one untrained panelists, aged between 18 and 69 years, voluntarily participated in an affective evaluation assessing acceptance and purchase intention of sandwich bread formulations containing varying concentrations of buriti and acuri oils, alongside a control sample formulated with soybean oil. The experimental design employed a simplex centroid mixture approach to systematically investigate the effects of lipid substitution levels on bread sensory properties. Substitutions of soybean oil with buriti or acuri oils were conducted at 0% (control), 25%, 50%, 75%, and 100%, enabling the evaluation of dose-dependent responses. Acceptance testing was performed using a structured 9-point hedonic scale, anchored by “like extremely” (9) and “dislike extremely” (1) [28]. Participants were recruited via convenience sampling, provided informed consent, and were presented with uniformly sliced bread samples served monadically and coded with randomized three-digit identifiers to prevent bias [29]. The consumer evaluation took place in a sensory laboratory in individual booths using a hedonic taste sheet. Sensory attributes assessed included overall acceptance, flavor, color, and texture. Additionally, participants reported purchase intention using a 5-point Likert-type scale ranging from “definitely would buy” to “definitely would not buy” [30]. The study protocol was approved by the Research Ethics Committee of the Federal Fluminense University under number CAAE: 61811522.6.0000.5243.

#### 2.8.2. Specific Volume

Bread samples were subjected to specific volume analysis (cm^3^/g), which is defined as the ratio between the displaced volume of the bread (cm^3^) and its weight (g), according to the rapeseed displacement method 10-05.01 [31].

#### 2.8.3. Colorimetry

The color of the oil and bread samples was measured using the CIELAB color space system, as proposed by the Commission Internationale de l’Éclairage (CIE) in 1971. In the breads, color measurement was performed only on the crumb. The colorimetric parameters L*, a*, and b*, in addition to the total color difference (ΔE) were measured using a Delta Color^®^ portable colorimeter. In this system, L* represents lightness (ranging from 0 = black to 100 = white), while a* and b* represent the chromaticity coordinates, with +a* indicating red, −a* green, +b* yellow, and −b* blue. Measurements were performed using i7 Gold 1.0.5.9 software integrated with the device.

#### 2.8.4. Texture Profile Analysis (TPA)

The texture profile analysis (TPA) of the bread crumb was performed using a TX-700 texture analyzer (Lamy Rheology Instruments, Tecnal, Brazil), following the AACC Method 74-09 [31]. The loaves were sliced and cut into 1.5 cm cubes, with the crust discarded. Each cube was subjected to two consecutive compression cycles using a 20 mm cylindrical probe, under a load of 40 g, with 50% deformation and a test speed of 0.5 mm/s. The parameters evaluated included hardness (N), springiness, adhesiveness (mJ), chewiness (mJ), gumminess (N), and cohesiveness. Nine measurements were performed for each treatment.

#### 2.8.5. Principal Component Analysis (PCA)

Data for the principal component analysis (PCA) were obtained from sensory evaluations using the hedonic scale, as well as from instrumental measurements of bread texture and crumb color. The variables associated with these attributes, together with the texture responses, were randomized and processed using Statistica 2020 software (TIBCO Software Inc., Data Science Workbench, version 14, Cloud Software Group, Palo Alto, CA, USA). The selected dataset was used to generate a two-dimensional (2D) projection of the variables on the factor plane. Two principal components were retained, accounting for more than 60% of the explained variance. Clusters were delineated by ellipses, with a significance coefficient set at 0.95.

#### 2.8.6. Fourier Transform Infrared Spectroscopy (FT-IR)

The infrared spectra of buriti and acuri oils, as well as of the breads formulated with these oils, were recorded using an ATR-FTIR spectrometer (IRTracer, Shimadzu, Kyoto, Japan) equipped with a diamond ATR module and a deuterated triglycine sulfate (DTGS) detector. All spectra were acquired at 25 °C over a wavenumber range of 4000–500 cm^−1^, with 32 scans and a spectral resolution of 4 cm^−1^ [32].

#### 2.8.7. Thermogravimetric Analysis (TGA)

The thermal properties of the bread were evaluated using thermogravimetric analysis (TGA) and differential thermal analysis (DTA) in a thermal analyzer (Q600, TA Instruments, Lindon, UT, USA), calibrated with indium. Between 3 and 7 mg of bread crumb was weighed and hermetically sealed in an aluminum crucible (Shimadzu, Barueri, SP, Brazil). An empty sealed crucible was used as the reference. The samples were heated from 30 to 600 °C and subsequently cooled, at a heating rate of 10 °C/min, under a nitrogen atmosphere with a flow rate of 50 mL/min. Thermograms were analyzed using Universal Analysis Software version 3.9 A (TA Instruments, New Castle, DE, USA).

### 2.9. Statistical Analysis

The data are presented as the mean ± standard deviation, analyzed in triplicate, and subjected to analysis of variance (ANOVA). Mean comparisons were performed using Tukey’s post hoc test at a 5% significance level in GraphPad Prism 9.0 (GraphPad Software Inc., San Diego, CA, USA). Sensory analysis and its correlation with instrumental measurements were conducted using Statistica 2020 software (TIBCO Software Inc., Data Science Workbench, version 14, Cloud Software Group, Palo Alto, CA, USA). The FTIR spectra curves were graphically analyzed using the OriginPro^®^ software version 2021 (OriginLab Corporation, Northampton, MA, USA).

## 3. Results and Discussion

### 3.1. Acuri Oil Extraction and Lipid Profile of Buriti and Acuri Oils

The yield of cold mechanical extraction of acuri oil from fresh pulp was 4.6%, whereas extraction from dried pulp yielded 6.8%. Higher yields have been reported in other studies employing solvent extraction methods; for instance, Lescano et al. [13] and Coimbra et al. [18] reported yields of 16.41% and 39.2%, respectively. While the use of elevated temperatures in solvent extraction (Soxhlet) facilitates the release of lipids, it has the disadvantage of potentially degrading thermosensitive bioactive compounds and poses environmental concerns [5].

The fatty acid profiles of buriti and acuri oils are presented in Table 2. Buriti oil is notably rich in oleic acid (78%), a monounsaturated fatty acid with well-documented benefits for cardiovascular health, immune system function, and metabolic regulation, in addition to anti-inflammatory and antitumor properties [12,33,34]. The high oleic acid content observed in buriti oil is consistent with findings from other studies, which reported similar values of 78%, 78.48%, 74.73%, and 77% [6,13,35,36]. In addition to its health-promoting effects, oleic acid exhibits high oxidative stability and strong resistance to thermal degradation, making it particularly suitable for technological applications in the food industry and culinary preparations [37]. According to Hernandez [38], the presence of antioxidants such as tocopherols can enhance the oxidative stability of vegetable oils, thereby extending their shelf life.

The lipid profile of acuri oil was also characterized by a predominance of oleic acid, followed by palmitic acid, with lauric and myristic acids, both medium-chain saturated fatty acids, present at levels higher than those previously reported in the literature. Although Lescano et al. [13] also observed significant levels of lauric and myristic acids (4.88% and 4.15%, respectively), they reported an oleic acid content of 51%, along with 20.5% palmitic acid and 12.7% linoleic acid. In contrast, Coimbra et al. [18] reported a higher oleic acid content of 67%, followed by 13% palmitic acid and 10% linoleic acid in acuri oil. Several studies have demonstrated that the fatty acid composition of vegetable oils can be influenced by a range of factors, including environmental conditions such as soil type, climate, and cultivar, as well as fruit ripening stage, storage duration, extraction method, and the type of solvent used [4,5,39].

Palmitic acid is the second most abundant fatty acid in both buriti and acuri oils (Table 2). While it is well established that the intake of palmitic acid, a long-chain saturated fatty acid, can elevate low-density lipoprotein (LDL) levels in the bloodstream, recent debates have questioned the direct association between its consumption and increased risk of cardiovascular disease [40]. A clinical study involving 4211 individuals aged 40 to 79 years found no significant association between the intake of saturated fatty acids and the incidence of major atherosclerotic cardiovascular events over a 10-year period [41]. It is also important to note that saturated fatty acids play a critical role as texture-modifying agents in food processing [42]. In this context, the use of buriti and acuri oils, which are rich in lipophilic bioactive compounds, becomes a more suitable alternative for bread formulations compared to hydrogenated vegetable fats, which are high in trans fatty acids.

The primary lipid ingredients used in the industrial formulation of sandwich bread are soybean oil and palm fat. While soybean oil is a good source of the essential fatty acid linoleic acid (C18:2 n-6), accounting for approximately 51%, it lacks significant levels of natural antioxidants and vitamin E and contains a relatively low proportion (22.8%) of monounsaturated fatty acids (MUFAs) [43].

According to a review by Abdullah et al. [44], which analyzed multiple randomized clinical trials, the dietary intake of MUFAs from various food sources has been associated with a reduced risk of cardiovascular disease and type 2 diabetes. Although palm fat presents a fatty acid profile similar to that of the vegetable oils evaluated in this study, comprising approximately 32 to 52% palmitic acid and 35 to 50% oleic acid, it is linked to social and environmental concerns. Between 2009 and 2019, approximately 20% of forested areas in the world were converted into oil palm plantations, a land-use change that contravenes the principles of Sustainable Development Goal 8, which advocates for inclusive and sustainable economic growth and decent work for all [37].

### 3.2. Total Phenolics Compounds, Phenolics Profile, and Antioxidant Activity

Phenolic compounds are well recognized for their antioxidant activity, which is associated with numerous health benefits, including the prevention of cardiovascular and neurodegenerative diseases, metabolic disorders, autoimmune conditions, and certain types of cancer. These properties highlight their potential for applications in food, cosmetics, and pharmaceuticals [7,39,45]. In vegetable oils, the most commonly occurring phenolic compounds are phenolic acids. The total phenolic content measured in buriti and acuri oils was 98.20 µg GAE/g and 67.18 µg GAE/g, respectively, values higher than those reported for flaxseed (24.49 ± 3.57 µg CAE/g), coconut (8.92 ± 0.66 µg CAE/g), sunflower (11.60 ± 2.36 µg CAE/g), and avocado oils (64.33 ± 3.57 µg CAE/g) [46]. The phenolic content in buriti oil was consistent with previous findings by Speranza et al. [33], who reported 107 µg GAE/g in oils from fruits collected in Amazonas, and by Silva et al. [47], who found 120 µg GAE/g in samples from Pará. In contrast, the phenolic content in acuri oil (67.18 µg GAE/g) was significantly higher than the values reported by Lima et al. [2] and Coimbra et al. [18], which were 0.02 µg GAE/g and 0.002 µg GAE/g, respectively. Although most phenolic compounds are hydrophilic in nature, some are present in the lipid fraction of oils.

Phenolic compounds are often responsible for key sensory attributes of vegetable oils, including astringency, bitterness, color, and aroma. However, their biological properties also play a critical role in their consideration as food ingredients. Among the most notable biological effects are their antioxidant, anti-inflammatory, and anticancer activities, as well as their ability to reduce LDL cholesterol levels [47,48]. In addition, phenolics contribute significantly to the oxidative stability of vegetable oils and are the primary bioactive compounds in olive oil. Similar to olive oil, the phenolic profile of oils extracted from palm fruits can be influenced by a variety of factors, such as soil type, cultivar, climate, and geographical origin [49]. Therefore, characterizing the phenolic composition of an oil is essential for understanding its nutritional and physicochemical properties. In buriti oil, seven phenolic compounds were identified, including quercetin, catechin, and various phenolic acids (Table 3).

The antioxidant activity of these oils and their potential in preventing chronic diseases are commonly associated with the presence of polyphenols, tocopherols, and carotenoids, often exhibiting synergistic effects [33]. In this study, buriti oil exhibited a notably high ABTS radical scavenging capacity (788.85 µmol TE/g), surpassing the 17.58 µmol TE/g reported by Marcelino et al. [45]. Acuri oil showed an ABTS value of 298.33 µmol TE/g, also higher than that previously reported by Lima et al. [2], which was 161.70 µmol TE/g. These results highlight the strong radical scavenging potential of both oils, suggesting their relevance as functional ingredients for the food industry. To date, no reports have been found in the literature regarding the antioxidant activity of acuri pulp oil using ABTS and DPPH assays. Despite its lower ABTS value compared to buriti oil, likely due to its lower content of carotenoids, tocopherols, and total phenolics, acuri oil still demonstrated a considerable antioxidant capacity (298.33 µmol TE/g).

Although triterpenes are commonly found in various parts of palm species, their presence in pulp oils is relatively rare. Nevertheless, a prominent peak corresponding to betulinic acid, a pentacyclic triterpene with known anti-inflammatory, antimicrobial, and anticancer properties, was detected in acuri oil, although not quantitatively determined. Betulinic acid has shown promising effects in inducing apoptosis in cancer cells [50,51], and its detection in acuri oil opens avenues for further clinical research on its potential as a bioactive food compound. Taken together, these findings reinforce the functional value of buriti and acuri oils as rich sources of phenolic compounds and natural antioxidants. Their complex composition and bioactive potential support their application as health-promoting ingredients in the development of functional foods, while also warranting further investigation into their therapeutic benefits and mechanisms of action.

### 3.3. Tocols Profile

In addition to phenolic compounds and carotenoids, tocols also play a crucial role in the oxidative stability of vegetable oils. These compounds function as potent antioxidants across both food matrices and biological systems, in addition to exhibiting vitamin E activity through their role as lipid-soluble chain-breaking agents that protect cellular membranes from oxidative damage [18]. Vitamin E is a fat-soluble vitamin composed of a group of eight isomers: α-, β-, γ-, and δ-tocopherols and α-, β-, γ-, and δ- tocotrienols. In the present study, buriti oil exhibited a total tocol content of 129.54 mg/100 g, a value comparable to that reported by Serra et al. [4] (151.10 mg/100 g). However, while Serra et al. [4] identified β-tocopherol (76.19 ± 1.20 mg/100 g) as the predominant isomer over α-tocopherol (45.15 ± 0.93 mg/100 g), our results showed a different distribution pattern (Table 3).

The total tocol content of buriti oil was higher than that of commonly used edible oils such as soybean (102.17 mg/100 g), corn (89.60 mg/100 g), and sunflower oils (68.18 mg/100 g), which are frequently employed in bread formulations. Acuri oil presented a total tocol content of 49.9 ± 1.43 mg/100 g, which is higher than that reported for peanut oil (34.08 mg/100 g) [52]. Considering that the recommended daily intake for an adult is 15 mg/day, consuming less than 100 g of buriti or acuri oil would be sufficient to meet this requirement [53]. In both buriti and acuri oils, α-tocopherol was the predominant isomer (Table 4). This compound is recognized for its highest biological activity due to its ability to scavenge free radicals and protect polyunsaturated fatty acids from oxidative degradation [54].

Nevertheless, emerging evidence highlights the superior bioactivity of tocotrienols, not only as antioxidants for lipids but also for their anticancer properties attributed to their stereospecific molecular structure, particularly the presence of double bonds in the C16-alkyl side chain [55]. These structural features enhance their ability to penetrate lipid-rich tissues, such as the brain and liver, allowing tocotrienols to exert more potent antioxidant effects compared to tocopherols [56]. Notably, acuri oil showed a significant α-tocotrienol content of 12.39 mg/100 g.

The high levels of lipophilic bioactive compounds in buriti and acuri oils, including tocopherols, tocotrienols, and carotenoids, support their classification as functional ingredients with promising applications in the development of health-promoting foods. These compounds exhibit antioxidant and anti-inflammatory properties and play a preventive role against chronic and neurodegenerative diseases [57].

### 3.4. Carotenoids Profile

Carotenoids are lipophilic pigments characterized by a structure rich in conjugated double bonds, which confer high chemical reactivity and enable light absorption in the visible range of the electromagnetic spectrum. These pigments exhibit a color spectrum ranging from yellow to red [24,58]. Carotenoids are widely recognized for their provitamin A activity, and more recent studies have investigated their protective effects against macular degeneration, rheumatoid arthritis, cognitive decline, and depression [25,45].

Due to their high concentration and structural diversity of carotenoids, buriti and acuri enable efficient release and solubilization of these compounds into the oils extracted from their pulps. This facilitates the incorporation of carotenoids into the lipid matrix, enhancing both the nutritional value and oxidative stability of the resulting oils. The carotenoid profile of acuri and buriti oil includes lutein, astaxanthin, β-carotene, and α-carotene, both of which are key precursors of vitamin A due to their high conversion efficiency to retinol. In both oils, β-carotene is the predominant carotenoid [25,45].

The total carotenoid content in buriti oil reached 1548.70 µg/g, which is higher than the values reported by Pereira et al. [58] (999.60 µg/g) and Kohn et al. [59] (722.72 µg/g) [58,59]. This variation may be attributed to climatic conditions, extraction techniques, or storage conditions. In an assessment of the total carotenoid content in 50 buriti oil samples collected from different municipalities in the state of Pará, Silva et al. [60] reported values ranging from 308.09 to 1898.92 µg/g. Remarkably, the present study found that β-carotene alone constituted 1476.50 µg/g, corresponding to nearly 95% of the total carotenoid content in buriti oil, underscoring its dominance within the carotenoid profile (Table 3). This value exceeds those previously reported in the literature, which ranged from 19.31 to 461.42 µg/g [56,59,61]. The high β-carotene content of buriti oil makes it particularly effective in mitigating oxidative stress, thereby contributing to the prevention of chronic and inflammatory diseases, retinal disorders, and supporting cellular regeneration. Furthermore, the oil’s notable antimicrobial and anti-inflammatory properties render it a promising candidate for topical applications aimed at the treatment of dermatological conditions. In addition, its compositional characteristics support its use as a functional ingredient in diverse food formulations [45,62].

Acuri oil exhibited a total carotenoid content of 168.10 µg/g and a β-carotene concentration of 112.78 µg/g, values that are consistent with those reported by Coimbra et al. [18], who found 240 µg/g of total carotenoids. Although Lima et al. [2] reported a higher total carotenoid content (394.8 µg/g), the β-carotene level was lower (65.3 µg/g) compared to the present study. An in vitro experimental study demonstrated the anti-inflammatory potential associated with the carotenoid content and antioxidant capacity of acuri pulp oil [7]. As illustrated in Table 4, other carotenoids also contribute to the carotenoid profile of acuri oil. According to the comprehensive review conducted by Aziz et al. [63], xanthophylls such as lutein and astaxanthin, which are present in acuri oil, have well-documented antioxidant properties and exert protective effects against neurological, immunological, allergic, and ophthalmological disorders, in addition to pharmacological interactions with β-carotene. These findings support the potential application of buriti and acuri oils as valuable dietary sources of carotenoids.

### 3.5. Bioacessibility of Carotenoids

Carotenoid bioaccessibility is defined as the fraction of dietary carotenoids liberated from the food matrix within the gastrointestinal tract during digestion, thereby becoming available for intestinal absorption [64]. It serves as an indicator of the nutritional quality of dietary carotenoids, particularly considering that humans are unable to synthesize these compounds endogenously [65]. Although the bioaccessibility of carotenoids in fruits and vegetables is generally low, mainly due to their limited lipid content, it is significantly enhanced in oils, as the efficiency of micelle formation during digestion is directly related to the proportion of fatty acids released during lipid hydrolysis. Thus, carotenoid-rich oils and fats are expected to promote increased mixed micelle formation and, consequently, greater bioaccessibility [66]. However, this behavior may be influenced by several factors, including fatty acid chain length, lipid droplet size, degree of unsaturation, and the surface area available for lipolytic enzyme activity [8,67].

Structurally, carotenoids are classified into two major groups: carotenes (e.g., α-carotene, β-carotene, and lycopene) and xanthophylls (e.g., lutein, bixin, β-cryptoxanthin, and astaxanthin). It is well established that carotenes are more efficiently micellarized in the presence of monounsaturated fatty acids (MUFA) and long-chain triglycerides, whereas xanthophylls are preferentially micellarized with saturated fatty acids and medium-chain triglycerides, which facilitates their subsequent absorption during digestion [65,68]. Overall, xanthophylls exhibit higher bioaccessibility than carotenes [68,69]. This pattern is corroborated in the present study, as lutein and astaxanthin demonstrated markedly higher recovery rates compared to α- and β-carotene, further supporting the hypothesis that micellization efficiency is strongly influenced by carotenoid polarity and the fatty acid composition of the lipid matrix.

Yuan et al. [70] demonstrated that β-carotene bioaccessibility was significantly higher in emulsions rich in MUFA compared to those containing saturated fatty acids. This finding is consistent with the present study, which observed β-carotene recovery rates of 53% in buriti oil and 67% in acuri oil. Considering that xanthophyll absorption is enhanced in the presence of saturated fatty acids, as reported by Failla et al. [71], the high palmitic acid content in both oils likely contributed to the excellent bioaccessibility of lutein and astaxanthin [71]. Supporting this hypothesis, Fernandes et al. [68] reported increased xanthophyll bioaccessibility, particularly of lutein, in microalgae containing higher levels of saturated fatty acids [68].

Moreover, MUFA-rich lipids, such as those found in buriti and acuri oils, are capable of forming micelles with a greater capacity for carotenoid solubilization, resulting in bioaccessibility levels two to three times higher than those observed with polyunsaturated fatty acid (PUFA)-rich or saturated fat-based oils [67,72]. Regardless of lipid type, the presence of dietary fats is essential for carotenoid bioaccessibility, as they promote the formation of mixed micelles through the solubilization of carotenoids in the lipid droplets of the gastric emulsion, while also stimulating bile salt and lipase secretion [67,69].

It is also important to note that during the oral, gastric, and intestinal phases of digestion, carotenoids may undergo structural isomerization from the all-trans to the cis configuration, the latter being more bioaccessible. However, cis-isomers may not have been detected using the HPLC method employed in the present study [68]. In this study, acuri oil showed a carotenoid recovery rate of 74%, while buriti oil reached 54%, with β-carotene exhibiting the greatest loss during in vitro digestion compared to the other carotenoids present in both oils (Table 5). These results are considerably higher than those reported in previous studies. Santos et al. (2023) found less than 2% recovery of carotenoids in crude tucumã oil, while Pinho Junior et al. (2025) reported only 0.5% recovery of α- and β-carotene in palm oil [8,66]. Similarly, Mohan et al. [73] reported β-carotene bioaccessibility of 8.7% in red palm oil. To date, no other studies have specifically evaluated the bioaccessibility of carotenoids in buriti and acuri oils [73].

### 3.6. Sensory Analysis of Breads

The results of the sensory evaluation of bread prepared with buriti and acuri oils, alongside the control bread formulated with soybean oil, are presented in Table 6. The products were assessed for color, flavor, texture, and overall acceptance. In general, most participants assigned scores above 7 on the 9-point hedonic scale, corresponding to the categories “liked moderately,” “liked very much,” and “liked extremely”, for all formulations evaluated, indicating a high level of consumer acceptance [28]. Among the sensory attributes analyzed, color emerged as the most influential factor in product acceptance. This finding aligns with previous studies highlighting color as a key determinant in consumer purchasing decisions and in the initial perception of food quality [74]. In this context, the natural carotenoids present in buriti and acuri oils played a relevant role in the visual appeal of the breads, as color was the only attribute that showed a statistically significant difference when compared to the control bread (BB0 and AB0).

Although a slight increase was observed in the mean hedonic scores for the formulations containing the tested oils, most samples did not show statistically significant differences (*p* ≤ 0.05) in the other evaluated attributes. This can be regarded as a positive outcome, as it suggests that the incorporation of buriti and acuri oils does not negatively affect consumer acceptance. On the contrary, it maintains a level of acceptance comparable to that of conventional sandwich bread, with which consumers are already familiar. Similar findings were reported by Halim et al. [75] in an acceptance test of gluten-free cakes enriched with carrot powder. Although the product exhibited a slightly higher overall acceptance score (7.40) compared to the control (7.10), no statistically significant difference was observed in color, despite the elevated carotenoid content from the added carrot.

For the purchase intent evaluation, a structured 5-point scale was used. The mean score for bread with buriti oil was 4.16, while bread with acuri oil scored 4.23. In both cases, more than 80% of participants indicated that they would “probably buy” or “definitely buy” the product. Aquino et al. [76] formulated cookies by replacing 15% of the soybean oil in the original recipe with laboratory-extracted and refined buriti oil. In a 7-point hedonic sensory evaluation conducted with children aged 7 to 10 years, the average overall acceptance score was 5. No significant difference in flavor was observed between the cookies containing 15% buriti oil and the control samples, suggesting that the inclusion of buriti oil has a minimal impact on the flavor profile of the final product [76].

### 3.7. Total Phenolics Compounds and Antioxidant Activity of Breads

**Figure 1:**
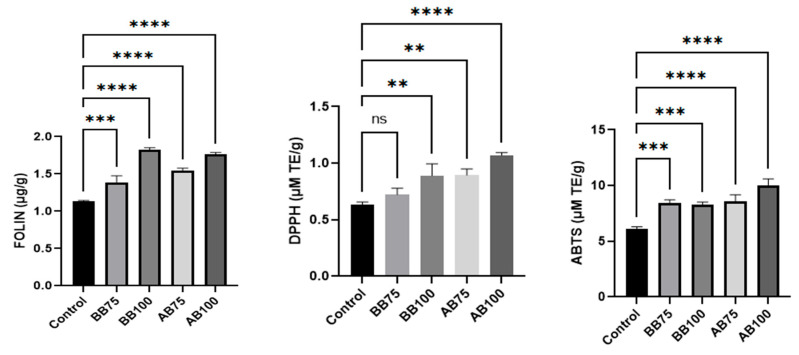
Total phenolic compounds and antioxidant activity of bread. Asterisks indicate significant differences (** *p* ≤ 0.05; *** *p* ≤ 0.01; **** *p* ≤ 0.001) and ns indicate no significant differences (*p* ≤ 0.05) according to Tukey’s *t*-test. ANOVA was performed only between the samples of bread with buriti oil (BB) and the control and between samples of bread with acuri oil (AB) and the control. No analysis of variance was performed between the breads with different oils.

The antioxidant activity of the control bread and formulations in which 75% and 100% of the soybean oil was replaced by buriti or acuri oils is presented in Figure 1. A significant difference was observed between the bread made with both oils and the control, with a noticeable increase in total phenolic content as the proportion of the alternative oils increased. This finding suggests that the bioactive compounds present in buriti and acuri oils, particularly phenolics, were retained during the baking process.

Comparable trends have been reported in the literature. For instance, white bread made with refined flour typically contains around 0.87 µg GAE/g of total phenolics [77]. When sunflower oil was replaced by pumpkin seed oil, rich in carotenoids, tocopherols, and flavonoids, Nilova et al. [78] observed a 115% increase in antioxidant activity as measured by the DPPH assay. Similarly, Yunusa et al. [79] reported an almost tenfold increase in DPPH radical scavenging activity when margarine was substituted with sweet orange seed oil in bread formulations.

In the present study, antioxidant activity generally followed the same trend as total phenolic content. However, an exception was observed in the bread containing 75% buriti oil, which did not differ statistically from the control in the DPPH assay. These results indicate that phenolic compounds, together with carotenoids and tocopherols, contribute synergistically to the antioxidant potential of the breads.

### 3.8. Instrumental Analysis of Breads

#### 3.8.1. Specific Volume

The specific volume of bread tends to increase with the rising saturation level of fatty acid chains present in the formulation. This effect is attributed to the behavior of saturated fats during baking, as they melt upon heating and promote the proper expansion of gas cells, thereby facilitating greater gas retention and contributing to a more aerated crumb structure [80]. In this context, bread formulated with buriti and acuri oils exhibited higher specific volumes as the substitution levels increased, which was directly associated with lower firmness values, as shown in Table 7.

In general, the incorporation of lipids enhances the specific volume of bread up to a certain threshold, beyond which adverse effects may occur. Ropciuc et al. (2022), for instance, reported that hemp oil supplementation at concentrations between 5% and 10% improved technological quality, including higher specific volume, while additions above 10% resulted in a decline in this parameter [81]. Similarly, Mikolasova et al. (2022) found that the inclusion of just 1.5% sunflower oil increased the specific volume of bread from 3.30 cm^3^/g (control sample) to 4.39 cm^3^/g, demonstrating the positive impact of low concentrations of vegetable oils on bread structure [82].

Although textural and volumetric properties may differ significantly between gluten-containing and gluten-free breads, research has shown consistent effects of lipid addition across both systems. Mancebo et al. (2017), for example, observed a substantial increase in the specific volume of gluten-free bread supplemented with vegetable oils compared to lipid-free controls [83]. The authors also noted a direct correlation between increased specific volume and reduced hardness, suggesting that the presence of oil contributes to softer bread textures. Zhang et al. (2021) further confirmed that the addition of soybean, corn, or rapeseed oils significantly enhanced dough specific volume and resulted in softer textures and more homogeneous crumb structures [84].

#### 3.8.2. Colorimetry of Oils and Breads

The colorimetric results of the oils, based on the CIELab system, are presented in Table 8. Soybean oil, used as the reference standard, exhibited higher L* values compared to buriti and acuri oils, which reflects its lighter and more translucent appearance. Both buriti and acuri oils showed darker coloration than soybean oil, as evidenced by their lower L* values, an indicator of lightness on a scale from 0 (black) to 100 (white). This difference is likely attributed to the presence of carotenoid pigments, as well as potential suspended solids derived from the fruit pulp.

The a* and b* parameters represent the chromaticity coordinates of the samples. Positive a* values, observed in both acuri and buriti oils, indicate a tendency toward red hues, while positive b* values suggest a shift toward yellow, in contrast to blue tones. Notably, buriti oil displayed higher b* and lower a* values than acuri oil, indicating a more intense yellow hue and less pronounced red coloration.

These findings are consistent with those reported by Marcelino et al. [45], who attributed the characteristic yellow color of buriti oil to its high β-carotene content.

The colorimetric results of the control bread and the samples formulated with buriti and acuri oils at four levels of substitution are presented in Table 8. Despite the chromatic differences between the oils, the color behavior of the breads showed particular patterns. Although statistically significant differences in L* values were observed between the control and the bread containing buriti and acuri oils, the values remained relatively close, suggesting that the incorporation of these oils did not substantially alter the overall appearance of the breads. Notably, the bread made with 100% buriti oil exhibited a darker color compared to the control. In contrast, bread formulated with acuri oil became progressively lighter with increasing substitution levels, which may be attributed to the markedly higher carotenoid content in buriti oil compared to acuri oil (Table 4).

Furthermore, breads prepared with both oils exhibited an increase in a* values (indicating a more reddish hue) and b* values (more yellowish hue) as the oil concentration increased. This effect is likely due to the high levels of carotenoids in buriti and acuri oils, which intensify the final product’s coloration.

#### 3.8.3. Texture Profile Analysis (TPA)

Lipids play multiple roles in baking, functioning not only as an energy source and carrier of bioactive compounds but also as key modulators of bread texture, volume, and shelf life. Their plasticity, determined by the ratio between solid and liquid lipid fractions, has a direct impact on dough structure by enhancing softness, chewability, and crumb staling delay. This is primarily due to the formation of starch–lipid complexes that inhibit retrogradation [83,84,85]. Triglycerides rich in saturated fatty acids confer greater plasticity to the lipid phase, facilitating the development of a more stable protein network during baking, which ultimately results in softer and more voluminous loaves [82].

In Brazil, soybean oil remains the predominant lipid source in approximately 67% of packaged bread formulations, followed by margarine, hydrogenated vegetable fats, or other vegetable oils [86]. This preference is largely driven by its wide availability, low cost, and satisfactory technological performance. However, soybean oil, rich in polyunsaturated fatty acids, exhibits low oxidative stability and a reduced content of bioactive compounds, thereby limiting its nutritional potential [87].

In the present study, substituting soybean oil with buriti and acuri oils significantly influenced the textural parameters of the bread, particularly reducing firmness and chewiness in samples formulated with 100% acuri oil (Table 9). This effect is likely related to the high saturated fatty acid content of acuri oil (54.23%), which enhances plasticity, improves oxidative stability, and may extend product shelf life. A similar trend was observed in bread made with buriti oil, which, despite containing a lower proportion of saturated fatty acids (19.10%), also led to a significant reduction in firmness and chewiness compared to the control, indicating a softer crumb structure (Table 9). However, elasticity was reduced in bread with higher concentrations of buriti oil, suggesting a more compact texture and reduced resilience after compression. Although lower elasticity is not desirable in all bread types, it is generally acceptable in sandwich breads, which naturally possess a less elastic crumb.

Regarding cohesiveness, a parameter related to crumb fragmentation, a slight but significant increase was observed in bread containing buriti oil, whereas no significant effect was found in those formulated with acuri oil. This suggests that the type of oil may have a limited influence on this attribute.

Although no previous studies were found investigating the use of buriti or acuri oils in bread formulations, instrumental texture parameters reported for other types of breads and lipids may serve as useful comparisons. For example, Zhang et al. [84] reported an increase in specific volume and a reduction in firmness in Chinese steamed breads when vegetable shortening was partially replaced with oleic acid-rich peanut oil (46% oleic acid) [84]. In contrast, substitution with soybean oil led to lower viscosity and elasticity, rendering it a less suitable alternative for improving dough performance. Additionally, Gerits et al. [87] demonstrated that unsaturated lipids are capable of interacting with gluten proteins, particularly glutenins, thereby enhancing the viscoelastic network and improving the rheological properties of wheat doughs. It is important to note, however, that gluten-related characteristics were not assessed in the present study [87].

Beyond their effects on texture, lipids also contribute to the development of volatile compounds responsible for flavor and aroma. Wu et al. [88] observed increased levels of aldehydes, ketones, and furans in sourdough breads enriched with corn oil, further supporting the functional role of lipids in sensory quality and consumer acceptance.

Taken together, these findings highlight the potential of buriti and acuri oils as promising alternatives to soybean oil in bakery applications. Their incorporation may improve both technological and sensory attributes of bread while also offering added nutritional value and supporting sustainable supply chains based on native Brazilian fruits.

#### 3.8.4. Principal Components Analysis (PCA) of Color and Texture Evaluations

**Figure 2:**
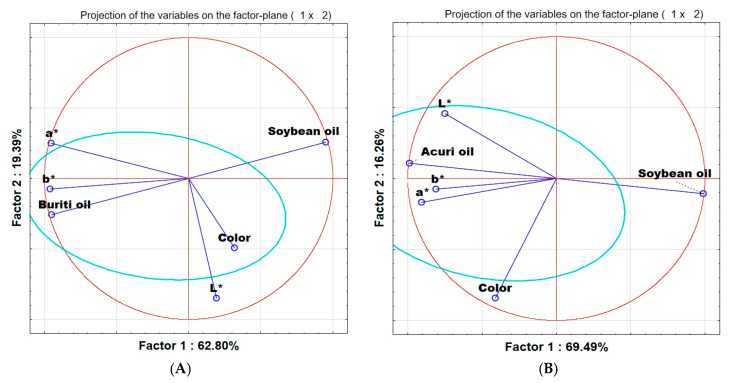
Principal component analysis (PCA)—sensory analysis and instrumental analysis. The color parameters a*, b*, and L* were obtained using the CIELab* color space. The point labeled “Color” corresponds to the sensory analysis of color perception. (**A**) compares buriti oil bread and soybean oil bread. (**B**) compares acuri oil bread and soybean oil bread.

The principal component analysis of the sensory and instrumental evaluations of the control breads and those containing buriti and acuri oils is presented in Figure 2A,B. The control breads are represented by the vector “Soybean oil,” while the terms “buriti oil” and “acuri oil” refer to the native oils used in the formulations.

Principal component analysis (PCA) shown in Figure 2A, comparing buriti oil breads (BB) and the control bread (soybean oil), indicates that sensory perception of color by consumers was more strongly associated with lightness (L*) than with the chromatic coordinates a* and b*. The BB samples are positioned in the lower-left quadrant, aligned with lower L* values and opposite to a* and b*, reflecting a darker appearance and reduced saturation in warm tones, consistent with their carotenoid-rich visual profile.

Similarly, in the PCA of breads containing acuri oil (AB), a weak correlation was observed between lightness and chromatic coordinates, while a* and b* displayed a positive correlation. In Figure 2B, however, the variable “Color” appears distinctly separated from the instrumental parameters, which may indicate that panelists gave greater weight to subjective aspects of color, such as saturation, vividness, or hue, not necessarily captured by L*, a*, or b* individually. The AB samples are aligned with lower a* and b* values and higher L* values, suggesting a paler, less saturated, but lighter color when compared, for example, to the BB samples.

**Figure 3:**
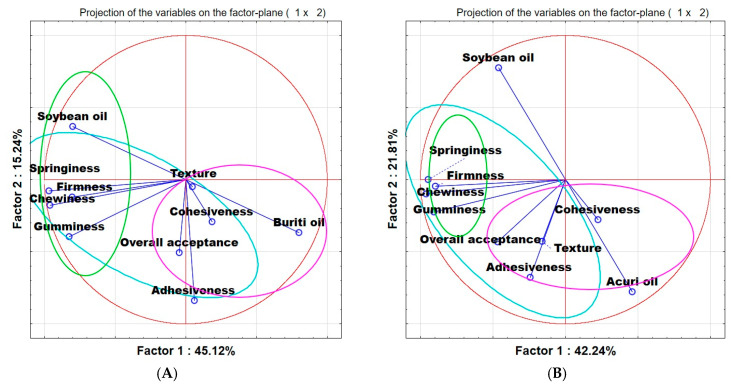
Principal component analysis (PCA)—sensory analysis and instrumental analysis. The points “soybean oil”, “buriti oil”, and “acuri oil” refer to the control bread, bread with buriti oil (**A**) and bread with acuri oil (**B**), respectively. “Texture” and “Overall acceptance” refer to sensory analysis, the other parameters were obtained from TPA.

With respect to bread texture, the principal component analysis (Figure 3) revealed that overall acceptability and sensory texture were more strongly associated with the use of buriti oil, whereas instrumental texture parameters showed no clear relationship with oil type. In the case of breads containing acuri oil, overall acceptability was also associated with the oil, but no correlation was found with instrumental measurements. These findings indicate that other factors, such as fermentation time, gluten strength, and flour characteristics, may have influenced the observed parameters, underscoring the need for further in-depth studies.

#### 3.8.5. Fourier-Transform Infrared Spectroscopy (FT-IR)

**Figure 4:**
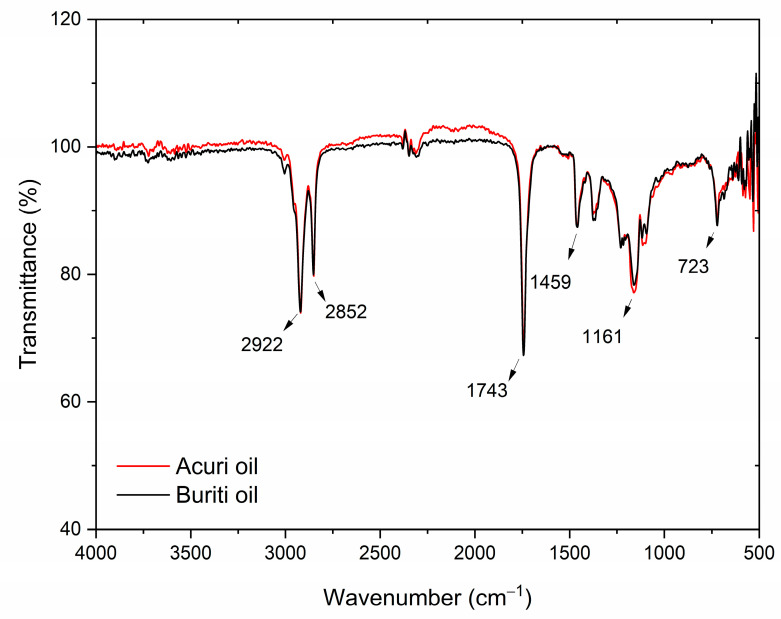
FT-IR spectra for acuri and buriti oils.

Infrared spectroscopy, often used in conjunction with other analytical techniques, is a standard method for determining the identity of oils and detecting potential adulteration. It also serves to confirm the predominant fatty acid composition of oils. Figure 4 shows the FT-IR spectra of crude buriti and acuri oils, which were found to be largely similar. The strong absorption bands observed at 1741 cm^−1^ for acuri oil and 1743 cm^−1^ for buriti oil are characteristic of carbonyl (C=O) stretching vibrations found in triglyceride esters, the primary constituents of vegetable oils [8,87,88,89]. These bands are consistent with the presence of long-chain fatty acids, as expected in buriti and acuri oils.

Additionally, the band observed at 1459 cm^−1^ is primarily associated with the bending vibration of methylene (–CH_2_–) groups within fatty acid chains and is a typical signature of lipid materials such as triglycerides. According to Silva et al. (2024), the spectral range between 1630 and 1755 cm^−1^, typical of carboxylic acids and ester, may also reflect contributions from phenolic acids in buriti oil [46]. The intense bands at 2922 cm^−1^ and 2852 cm^−1^ are attributed to asymmetric and symmetric stretching vibrations of CH_2_ groups, respectively, indicating the presence of saturated hydrocarbon chains in the oil molecules, corresponding to the palmitic acid content of both oils [89]. The absorption band at 3005 cm^−1^ corresponds to C–H stretching in =C–H (sp^2^) bonds, characteristic of unsaturated fatty acids and likely associated with oleic acid. Albuquerque et al. [90] reported the same band in the spectrum of pure oleic acid, and similar vibrational patterns have been observed in other vegetable oils commonly used in the food industry, such as soybean, palm, and palm kernel oils [46,90].

Moreover, multiple bands appearing in the region between 1100 and 1500 cm^−1^, especially the peak at 1161 cm^−1^, are possibly associated with molecular arrangements resembling those of triolein, indicating structural similarities between acuri and buriti oils and olive oil [91]. Previous studies have shown that buriti oil contains approximately 91.4% triacylglycerols, of which 28.8% is triolein, while olive oil contains around 32.5% triolein, suggesting that buriti oil may exhibit biological properties similar to those of olive oil [33]. Finally, the bands at 723 cm^−1^ are attributed to out-of-plane bending vibrations associated with cis-double bonds between carbon atoms, which characterize unsaturated fatty acids in their natural cis configuration.

**Figure 5:**
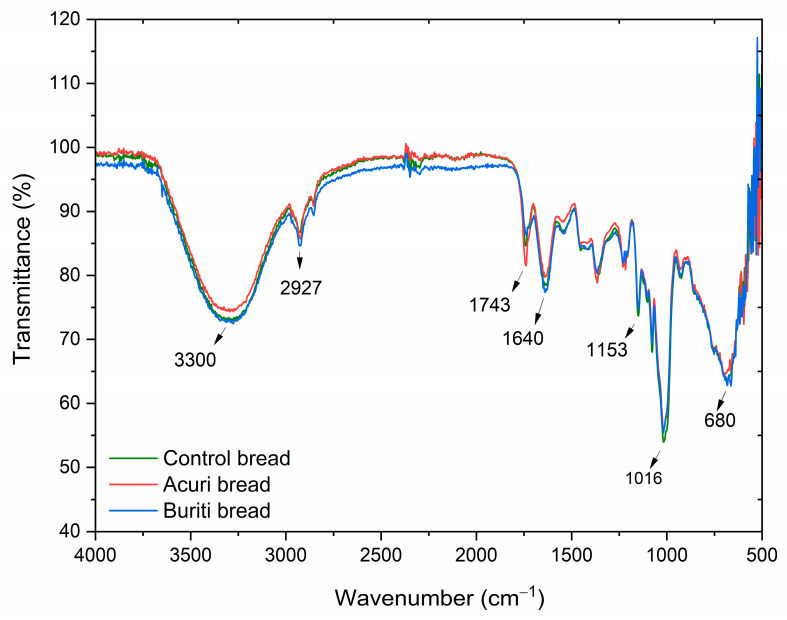
FT-IR spectra for control bread, BB100, and AB100.

The infrared spectra of control bread, BB100, and AB100 samples are shown in Figure 4 and Figure 5, respectively. The broad absorption band near 3300 cm^−1^ is attributed to the presence of water in the bread matrices. The bands at 2920 and 1743 cm^−1^ correspond to the C–H and C=O stretching vibrations of lipids, respectively, and are associated with the incorporation of vegetable oils into the dough formulations [92]. The overall overlap among the spectra suggests no major structural differences between the control, BB100, and AB100 samples. However, a slight reduction in the intensity of the 1743 cm^−1^ band was observed in the bread containing 100% buriti oil, which may be related to partial oxidation or degradation of fatty acid esters during breadmaking.

The absorption bands at 1153 and 1016 cm^−1^ are typical of starch transformations that occur during baking and subsequent cooling, corresponding to the (C–O–C) stretching of glycosidic ester bonds and asymmetric (C–O and C–C) stretching, respectively. In particular, the band at 1016 cm^−1^ is associated with starch retrogradation and reflects the recrystallization of amylopectin [93,94]. The superimposition of spectra at this wavelength across BB100, AB100, and the control suggests that lipid source substitution did not significantly affect starch retrogradation, the primary mechanism of bread staling. Likewise, no unique absorption bands were identified in the buriti or acuri bread samples that were absent in the control, indicating that the molecular structure of key functional groups remained unaffected by the lipid replacement. These findings suggest that the breads enriched with buriti and acuri oils preserved similar structural, visual, and sensory properties compared to the control, supporting their potential acceptability in commercial bakery applications, an outcome also supported by instrumental and sensory analyses.

#### 3.8.6. Thermogravimetric Analysis of Breads (TGA)

**Figure 6:**
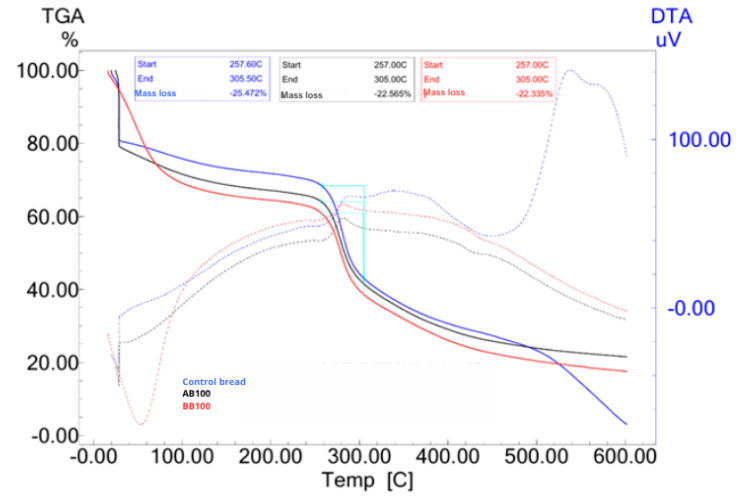
TGA/DTA control bread (blue line), BB100 (red line), and AB100 (black line). Solid lines represent TGA and dotted lines represent DTA.

Thermogravimetric analysis (TGA) was performed to assess the thermal stability of the control bread and the bread containing a 100% substitution of soybean oil with buriti (BB100) and acuri oil (AB100), within the temperature range of 30 °C to 600 °C. The TGA curves and differential thermal analysis (DTA) are shown in Figure 6. The first weight-loss stage, observed between 30 °C and 120 °C, corresponds to the evaporation of water retained in the bread matrix. The BB100 sample retained a higher amount of water in the bread compared to the other formulations, as evidenced by the smoother TGA curve with greater mass loss in the initial stage, and by the more pronounced endothermic peak observed in the DTA curve. These results suggest that bread formulated with native fruit oils exhibited greater water retention capacity, likely due to differences in the lipid matrix structure or specific interactions between lipids and starch components in the wheat flour [92,95].

The second weight-loss phase, observed between 257 °C and 305 °C in all three samples, is attributed to the thermal degradation of carbohydrates, proteins, and lipids. The BB100 and AB100 samples exhibited lower total mass loss (22.33% and 22.56%, respectively) compared to the control (25.47%), indicating greater thermal resistance that can be attributed to the presence of antioxidant bioactives from buriti and acuri oils. In the final degradation stage, near 500 °C, the TGA curves of the BB100 and AB100 samples (represented by red and black lines, respectively) end at higher residual mass levels compared to the control bread. This behavior highlights the presence of thermally stable compounds in the native fruit oils, possibly derived from antioxidant constituents, that persist even after bread baking.

Cheng et al. [96] reported that the addition of canola oil to white bread increased moisture retention as oil concentration increased. However, the thermal decomposition temperature of carbohydrates and proteins remained unchanged, indicating no significant influence of oil presence on this stage of thermal stability. Additionally, the authors observed an endothermic peak near 400 °C, associated with the degradation of unsaturated fatty acids, a feature that was also evident in the DTA curves of the control, BB100, and AB100 samples in the present study, where peaks appeared between 410 °C and 500 °C (Figure 6) [96]. Marcelino et al. (2022) attributed the decomposition peak observed between 276 °C and 479 °C in pure buriti oil to the presence of long-chain fatty acids [45].

The results clearly demonstrate that replacing soybean oil with buriti and acuri oils markedly influences the thermal stability profile of bread, affecting both moisture retention and the decomposition behavior of organic components. These modifications should be taken into account in industrial applications involving thermal processing or extended storage. Moreover, the findings reinforce the functional and technological potential of these regional oils as promising ingredients in bakery formulations.

## 4. Conclusions

This study highlights the promising potential of native Brazilian oils, specifically buriti and acuri oils, as functional ingredients in bread formulations. Despite the limited commercial exploitation of these fruits, their richness in bioactive compounds such as phenolics, tocopherols, and carotenoids underscores their agro-industrial relevance, particularly in the development of healthier and more sustainable food products. These bioactive constituents play a crucial role in mitigating lipid peroxidation and have been associated with protective effects against the onset and progression of chronic degenerative conditions, including diabetes, cardiovascular diseases, and various forms of cancer [2,4,56]. Of particular note is the high content of β-carotene, a potent vitamin A precursor, especially abundant in buriti oil.

Future studies should further advance the biochemical characterization of buriti and acuri oils, including detailed lipidomic profiling, to better understand their behavior under different processing and storage conditions. Investigations into bioaccessibility and bioavailability after digestion are also essential to substantiate health claims and optimize their application in food systems. Although processing may affect the stability of lipophilic bioactives such as carotenoids and tocopherols, the high bioaccessibility of these compounds in their native oil form reinforces their value for both raw and processed applications.

With regard to sandwich bread applications, the results demonstrated that replacing soybean oil with buriti or acuri oil improved crumb softness, as evidenced by the reduction in key textural parameters such as firmness and chewiness, in agreement with the high sensory acceptance observed. Importantly, the addition of naturally pigmented oils did not negatively affect sensory acceptance. The preservation of antioxidant activity in breads containing higher levels of native oils suggests that their functional properties are retained, at least in part, after baking. While no major differences were observed in the thermal behavior (TGA) or FTIR spectra of oil-enriched breads compared with the control, changes in fatty acid profiles resulting from substitution indicated enhanced thermal stability, an important attribute for extending shelf life and preserving nutritional quality.

Given the widespread consumption of bread across age groups and cultures, incorporating nutrient-rich oils from underutilized tropical fruits represents a strategic opportunity to meet the growing consumer demand for clean-label, health-promoting foods. Moreover, the inclusion of these oils supports bioeconomic development by adding value to Amazonian and Cerrado biodiversity and fostering sustainable supply chains rooted in local communities.

## Figures and Tables

**Table 1:** Bread formulations.

	Oil Substitutions in Bread Formulations				
Ingredients (g)	0%	25%	50%	75%	100%
Wheat flour	540	540	540	540	540
Water	286–308	286–308	286–308	286–308	286–308
Soybean oil	38	28.5	19	9.5	0
Buriti or acuri oil	0	9.5	19	28.5	38
Refined sugar	22	22	22	22	22
Dry yeast	9	9	9	9	9
Salt	11	11	11	11	11

**Table 2:** Fatty acid profile of buriti oil and acuri oil.

Fatty Acid (%)		Buriti Oil	Acuri Oil
Caprylic acid	C8:0	ND	0.57 ± 0.07
Capric acid	C10:0	ND	0.72 ± 0.04
Lauric acid	C12:0	0.02 ± 0.00	12.00 ± 0.54
Myristic acid	C14:0	0.06 ± 0.00	13.15 ± 0.23
Pentadecanoic acid	C15:0	0.04 ± 0.00	ND
Palmitic acid	C16:0	17.49 ± 0.57	24.87 ± 0.18
Palmitoleic acid	C16:1	0.15 ± 0.09	0.15 ± 0.00
Heptadecanoic acid	C17:0	0.07 ± 0.00	0.05 ± 0.00
Heptadecenoic acid	C17:1	0.05 ± 0.00	ND
Stearic acid	C18:0	1.30 ± 0.10	2.64 ± 0.03
Oleic acid	C18:1	78.25 ± 0.58	39.05 ± 0.42
Linoleic acid	C18:2	1.69 ± 0.01	6.20 ± 0.14
Linolenic acid	C18:3 n3	0.77 ± 0.01	0.21 ± 0.01
Linolenic acid	C18:3 n6	ND	ND
Eicosanoic acid	C20:0	0.08 ± 0.00	0.16 ± 0.00
Eicosapentaenoic acid	C20:5 n2	0.12 ± 0.00	0.07 ± 0.00
Tricosanoic acid fatty acids	C23:0	0.04 ± 0.01	0.07 ± 0.03
**Total saturated**		19.10 ± 0.68	54.23 ± 1.12
**Total unsaturated**		81.03 ± 0.69	45.68 ± 0.57
**Total fatty acids**		100.13 ± 1.3	99.91 ± 1.69

ND: not detected.

**Table 3:** Total phenolics compounds, phenolics profile, and antioxidant activity.

	Buriti Oil	Acuri Oil
DPPH (µMol Trolox/g)	25.21 ± 0.96	24.79 ± 0.24
ABTS (µMol Trolox/g)	783.85 ± 11.65	298.33 ± 4.71
TPC (µg/g)	98.90 ± 1.32	67.18 ± 0.11
o-Coumaric acid (µg/g)	14.35 ± 0.14	16.63 ± 0.04
p-Coumaric acid (µg/g)	17.32 ± 0.16	20.41 ± 0.04
Ferulic acid (µg/g)	12.74 ± 0.12	14.57 ± 0.02
Quercetin (µg/g)	14.37 ± 0.05	15.57 ± 0.01
Galic acid (µg/g)	14.48 ± 0.11	ND
HO benzoic acid (µg/g)	8.03 ± 0.06	ND
Catechin (µg/g)	17.61 ± 0.68	ND

ND: not detected.

**Table 4:** Carotenoids and tocols profile.

Tocols (mg/100 g)	Buriti Oil	Acuri Oil
α-Tocopherol	53.84 ± 1.89	18.15 ± 0.22
β-Tocopherol	34.24 ± 0.58	10.81 ± 0.64
γ-Tocopherol	41.46 ± 0.51	8.55 ± 0.18
α-Tocotrienol	ND	12.39 ± 0.39
Total	129.54 ± 2.98	49.9 ± 1.43
Carotenoids (μg/g)		
Bixin	ND	ND
Astaxanthin	13.76 ± 0.02	10.91 ± 0.09
Lutein	13.66 ± 0.03	11.13 ± 0.05
Zeaxanthin	ND	ND
α-Carotene	44.78 ± 0.59	33.24 ± 2.32
β-Carotene	1476.50 ± 9.74	112.78 ± 4.78
Total	1548.70 ± 8.54	168.10 ± 6.68

ND: not detected.

**Table 5:** Profile and bioaccessibility ^1^ of carotenoids after in vitro digestion.

	Buriti Oil	% Recovery	Acuri Oil	% Recovery
Astaxanthin	12.63 ± 0.02 *	91.78	10.93 ± 0.01	100
Lutein	12.75 ± 0.01 *	93.34	11.62 ± 0.01	100
α-Carotene	30.64 ± 0.03 *	68.42	26.42 ± 0.47 *	79.48
β-Carotene	789.60 ± 8.16 *	53.48	75.95 ± 0.96 *	45.18
Total	845.60 ± 8.21 *	54.3 ± 0.65	124.90 ± 1.20 *	74.4 ± 2.29

Bioaccessibility was calculated as the percentage of carotenoid concentration relative to the initial concentration shown in Table 4. Results are presented as the mean ± SD of triplicate analyses. Asterisks (*) indicate statistically significant differences between undigested and digested samples.

**Table 6:** Mean and standard deviation of the scores of the acceptance tests and purchase intention of breads added with buriti oil and acuri oil.

	Color	Taste	Texture	Overall Acceptance
BB0	6.94 ± 1.81 ^a^	7.23 ± 1.50 ^a^	7.62 ± 1.30 ^a^	7.35 ± 1.47 ^a^
BB25	7.78 ± 1.34 ^b^	7.20 ± 1.46 ^a^	6.89 ± 1.34 ^b^	7.51 ± 1.17 ^a^
BB50	7.79 ± 1.23 ^b^	7.00 ± 1.49 ^a^	7.06 ± 1.55 ^a^	7.27 ± 1.28 ^a^
BB75	7.64 ± 1.38 ^b^	6.84 ± 1.87 ^a^	7.04 ± 1.73 ^a^	7.46 ± 1.40 ^a^
BB100	7.85 ± 1.30 ^b^	7.20 ± 1.70 ^a^	7.37 ± 1.40 ^a^	7.56 ± 1.21 ^a^
AB0	6.80 ± 1.91 ^a^	6.93 ± 1.63 ^a^	7.11 ± 1.72 ^a^	7.04 ± 1.58 ^a^
AB25	7.53 ± 1.36 ^b^	7.06 ± 1.42 ^a^	6.81 ± 1.67 ^a^	7.32 ± 1.23 ^a^
AB50	7.10 ± 1.75 ^a^	7.25 ± 1.48 ^a^	6.77 ± 1.70 ^a^	7.15 ± 1.44 ^a^
AB75	7.64 ± 1.49 ^b^	7.43 ± 1.53 ^a^	7.41 ± 1.51 ^a^	7.64 ± 1.22 ^b^
AB100	7.38 ± 1.42 ^a^	7.16 ± 1,41 ^a^	7.10 ± 1.69 ^a^	7.38 ± 1.36 ^a^
Purchase Intention BB	4.16 ± 0.91			
Purchase Intention AB	4.23 ± 0.90			

Buriti oil (BB), acuri oil (AB). (BB0/AB0): 0% buriti or acuri oil; (BB25/AB25): 25% buriti or acuri oil; (BB50/AB50): 50% buriti or acuri oil; (BB75/AB75): 75% buriti or acuri oil; (BB100/AB100): 100% buriti or acuri oil. Lowercase letters indicate no significant differences (*p* ≤ 0.05) according to Tukey’s *t*-test.

**Table 7:** Specific volume.

	Control	BB25	BB50	BB75	BB100	AB25	AB50	AB75	AB100
Specific volume (cm^3^/g)	2.56 ± 0.04 ^a^	3.30 ± 0.10 ^a^	3.83 ± 0.14 ^b^	4.13 ± 0.10 ^c^	4.20 ± 0.19 ^cd^	2.99 ± 0.17 ^b^	3.13 ± 0.04 ^b^	3.30 ± 0.18 ^c^	4.10 ± 0.05 ^d^

Lowercase letters indicate no significant differences (*p* ≤ 0.05) according to Tukey’s *t*-test. ANOVA was performed only between the samples of bread with buriti oil (BB) and the control and between samples of bread with acuri oil (AB) and the control. No analysis of variance was performed between the breads with different oils.

**Table 8:** Colorimetric analysis of oils and breads.

	L*	a*	b*	ΔE
Soybean oil	67.19 ± 0.03 ^a^	−2.14 ± 0.38 ^a^	6.40 ± 0.22 ^a^	0.81 ± 0.07 ^a^
Buriti oil	41.75 ± 0.01 ^b^	13.14 ± 0.04 ^b^	2.27 ± 0.02 ^b^	28.14 ± 0.01 ^b^
Acuri oil	51.19 ± 0.01 ^c^	21.73 ± 0.04 ^c^	18.23 ± 0.07 ^c^	31.42 ± 0.05 ^c^
Control	65.79 ± 0.07 ^c^	2.16 ± 0.07 ^a^	17.12 ± 0.04 ^a^	7.29 ± 0.10 ^a^
BB25	65.35 ± 0.06 ^d^	7.59 ± 0.08 ^b^	35.59 ± 0.07 ^b^	12.73 ± 0.06 ^b^
BB50	66.88 ± 0.01 ^b^	8.70 ± 0.03 ^c^	47.09 ± 0.23 ^c^	20.08 ± 0.14 ^c^
BB75	74.79 ± 0.02 ^a^	7.65 ± 0.03 ^b^	44.87 ± 0.04 ^d^	19.85 ± 0.02 ^c^
BB100	61.74 ± 0.09 ^e^	12.06 ± 0.04 ^d^	53.00 ± 0.16 ^e^	24.45 ± 0.42 ^d^
AB25	63.72 ± 0.09 ^b^	6.00 ± 0.15 ^b^	25.18 ± 0.13 ^b^	6.45 ± 0.04 ^b^
AB50	66.85 ± 0.02 ^c^	6.08 ± 0.04 ^b^	29.89 ± 0.13 ^c^	8.92 ± 0.08 ^c^
AB75	67.85 ± 0.04 ^d^	8.14 ± 0.01 ^c^	27.66 ± 0.02 ^d^	8.81 ± 0.01 ^d^
AB100	67.94 ± 0.07 ^d^	8.91 ± 0.13 ^d^	28.08 ± 0.09 ^e^	7.89 ± 0.01 ^d^

Different lowercase letters within the same column indicate statistically significant differences (*p* ≤ 0.05) according to Tukey’s test.

**Table 9:** Texture profile analysis of bread.

TPA	Control	BB25	BB50	BB75	BB100	AB25	AB50	AB75	AB100
Firmness (N)	9.36 ± 0.98 ^a^	8.28 ± 1.21 ^ab^	8.45 ± 0.67 ^ab^	7.96 ± 0.69 ^bc^	7.27 ± 0.49 ^c^	8.14 ± 0.86 ^b^	6.31 ± 0.37 ^c^	7.01 ± 0.50 ^c^	6.99 ± 0.76 ^c^
Springiness	0.55 ± 0.03 ^a^	0.52 ± 0.12 ^a^	0.42 ± 0.06 ^b^	0.45 ± 0.10 ^b^	0.25 ± 0.06 ^c^	0.33 ± 0.07 ^c^	0.29 ± 0.05 ^c^	0.42 ± 0.06 ^b^	0.31 ± 0.07 ^c^
Adhesiveness (mJ)	0.00 ± 0.00 ^b^	0.09 ± 0.03 ^a^	0.10 ± 0.00 ^a^	0.10 ± 0.02 ^a^	0.10 ± 0.00 ^a^	0.10 ± 0.03 ^a^	0.10 ± 0.00 ^a^	0.20 ± 0.05 ^a^	0.10 ± 0.02 ^a^
Cohesiveness	1.24 ± 0.08 ^c^	1.52 ± 0.10 ^a^	1.45 ± 0.04 ^a^	1.40 ± 0.13 ^ab^	1.31 ± 0.07 ^b^	1.25 ± 0.10 ^c^	1.37 ± 0.07 ^c^	1.36 ± 0.15 ^c^	1.25 ± 0.12 ^c^
Gumminess	11.53 ± 0.79 ^a^	12.48 ± 1.11 ^a^	12.20 ± 0.62 ^a^	12.06 ± 1.16 ^a^	9.51 ± 0.45 ^b^	9.96 ± 0.64 ^b^	8.66 ± 0.81 ^bc^	9.13 ± 0.37 ^bc^	9.11 ± 0.32 ^c^
Chewiness (J)	6.31 ± 0.81 ^a^	6.54 ± 1.89 ^a^	5.19 ± 0.96 ^b^	5.43 ± 1.64 ^b^	2.39 ± 0.71 ^c^	3.38 ± 0.83 ^bc^	2.49 ± 0.42 ^d^	3.93 ± 0.28 ^b^	2.69 ± 0.57 ^cd^

Lowercase letters indicate no significant differences (*p* ≤ 0.05) according to Tukey’s *t*-test. ANOVA was performed only between the samples of bread with buriti oil (BB) and the control and between samples of bread with acuri oil (AB) and the control. No analysis of variance was performed between the breads with different oils.

## Data Availability

Data are contained within the article.

## Abbreviations

g	grams
µg	micrograms
mL	milliliters
AACC	American Association of Cereal Chemists
DPPH	2,2-difenil-1-picrilhidrazil
ABTS	2,2′-azino-bis (3-ethylbenzothiazoline-6-sulfonic acid)
